# Diseases of the Blood

**Published:** 1904-10-15

**Authors:** 


### DISEASES OF THE BLOOD.

E. L. Opie[1] has investigated the relation of eosinophile cells to nutrition. They are present not only in the blood, but also in the mucosa of the alimentary tract and of the air passages, in the lymphatic tissues and the spleen. They are produced from mononuclear cells in the marrow and discharged into the blood in numbers which vary periodically. Their numbers lessen when starvation takes place and increase when food is again given. Lovell Gulland[2] finds that to examine bone marrow satisfactorily it is best to rub up the marrow with a little salt solution of about ·9 per cent. strength and to make a film of the mixture, staining with Jenner's fluid. Leucocytosis in appendicitis is again shown to be a sign which it is difficult to interpret definitely. H. French[3] in 83 cases at Guy's Hospital found that while some cases where pus was present gave low counts, some where no pus was formed showed 20,000 and even 30,000 leucocytes. Still higher counts only occurred with pus, and an increasing number in successive counts was also a sign of pus, though this increase does not always occur. Dudgeon thought that the differential count was important as 80 or 90 per cent. of polynuclear cells were often seen if pus existed. L Opie[4] gives another paper on the increase of eosinophile cells produced by the administration of trichinæ to guinea pigs. The cells accumulate in the mesentery and lungs, and in the marrow there are corresponding changes. Under large doses and when death is approaching there is a diminution of the cells. Thus a mild infection increases them and a severe one destroys them. In another paper[5] he notes that the introduction of acutely fatal bacilli cause the disappearance of eosinophile leucocytes in a few hours from the peripheral circulation; more chronic forms lead to a gradual vanishing. After a non-fatal injection there is a temporary increase, but they rarely act as phagocytes. Eosin myelocytes appear in the blood a few hours after infection. On the whole bacteria attract eosinophiles from the bone marrow into the blood and thence to the site of inoculation. From Khartoum[6] the blood condition in cases of bilharzia disease and dracontiasis is reported. A high percentage of coarsely granular eosinophiles and of large mononuclears is found in all. In skin diseases[7] it appears that the generalisation that eosinophilia is common needs to be accepted with reserve. An examination of 90 cases revealed an excess of eosinophiles in 10 only, and in only four was it at all marked. The cases did not include one of pemphigus, however, with which it is said that eosinophilia is most often associated. In cyanosis, the red count is commonly very high. In a case[8] in which there were 8,500,000 red cells per cmm. and 93 per cent. of hæmoglobin in the capillary blood, blood taken from a small twig of the temporal artery gave 7,000,000 and 93 per cent., while that from a vein gave 10,000,000 and 115 per cent. There must, however, be a considerable error in one of the determinations of either the arterial or the capillary blood. The "volume-index"[9] is the fraction indicating the relation of the volume per cent. of the red cells to their numerical percentage. The volume occupied by the cellular elements when these are 5,000,000 per cmm. is taken as the standard and called 100. Capps estimates the volume with the Hæmocrit, an instrument which has so far met with little favour, as it is both difficult to use and, to obtain accurate results, it needs a specially prepared diluting fluid for each observation. Otherwise the variations of osmotic pressure in different bloods and at different times in the same individual will cause error. Capps, however, uses blood alone, taking care to avoid premature clotting by rapidity of working and scrupulous cleanliness. In pernicious anæmia it is shown that the heightened colour-index is due to the increased average size of the red cells. In normal blood the hæmoglobin "saturates" the protoplasm of the cells; "supersaturation" probably never occurs, and in pernicious anæmia the cells are not quite saturated. In certain secondary anæmias there is at first a low volume-index and colour-index; but later, a high volume and colour-index. In chlorosis the volume-index is diminished, but to a less extent than the colour-index (subsaturation); a fact which may prove of some value from a prognostic point of view is that a volume-index only slightly reduced indicates a speedy recovery, and a low volume-index is met with in cases which prove intractable under treatment. Colour-index gives no help in making a prognosis. The scientific grants committee of the British Medical Association has published a report[10] on the value of Hammerschlag's specific gravity method. The conclusions arrived at are that the results are uniform and reliable, both in health and disease, if the same hydrometer is used, but that there is always a slight error in the direction of excess. This can be obviated by the use of a specially-standardised hydrometer, or even by the application of a rough correction suggested by the observer. Though at first sight it would seem improbable that a good blood-film could be prepared on glass which has been ground rough by the use of emery paper, this is a method recommended by A. E. Wright.[11] Of course when oil or balsam is placed on the surface it is cleared, and the advantage is that the film is evenly spread, grouping of the red cells, and separation of the leucocytes towards the end at which the smear is finished, being to a great extent avoided. The grouping is not, as is usually said, due to grease on the slide, but to the smoothness of the surface. The same worker gives a detailed account of a method by which blood or other fluids may be "citrated" or otherwise diluted in a definite proportion, so that they are available not only for transmission to a laboratory, but can also be examined with a view to ascertaining the character and quantity of the albuminous constituents. The white cells probably possess a thermotactic property, and it is partly this perhaps that brings them to the seat of an inflammatory process. It has been shown that they move best at a temperature of 39°–39 5°, but they exhibit movement in some degree at a temperature as low as 20°–25°. Above 40° little movement is seen, and what little there is appears to be towards the regions of lower temperature. Recent experiments have shown (*Journal of Physiology*, vol. xxx., p. 1) that leucocytes increase regularly in numbers in the blood, and reach a maximum four hours after a meal. There is lymphocytosis, very regular both in degree and incidence, and an increase of the polymorphonuclears which is more variable. Removal of the spleen does not affect the leucocytosis of digestion.

1 Amer. Jour. Med. Sc., Feb. 2 Scottish Med. and Surg. Jour., June. 3 Brit. Med. Jour., May 14. 4 Amer. Jour. Med. Sc, March. 5 Ibid., June. 6 Lancet, Dec. 10. 7 Guy's Hosp. Rep., vol. lviii. 8 Lancet, Dec. 5. 9 Jour. Med. Research, Dec.; Med. Rec., March 26. 10 Brit. Med. Jour., Feb. 27. 11 Lancet, Jan. 22; July 9.

*(To be continued.)*

